# Transition between segregation and aggregation: the role of environmental constraints

**DOI:** 10.1038/srep32703

**Published:** 2016-09-07

**Authors:** Stamatios C. Nicolis, José Halloy, Jean-Louis Deneubourg

**Affiliations:** 1 Unit of Social Ecology Université Libre de Bruxelles, 1050 Bruxelles, Belgium; 2 Laboratoire Interdisciplinaire des Énergies de De main, Université Paris Diderot, Paris VII, France

## Abstract

Interactions between sub-groups (species, strains) have been reported in many species among many taxae. We propose a generic model based on earlier experiments accounting for both conspecific (or between individuals of the same strains) and heterospecific (or between strains) interactions. The model predicts different collective behaviours without any change of individuals’ algorithm as some key generic parameters such as the carrying capacity, the number of individuals involved and the strength of inter-attraction between sub-groups are varied. A key result is the possibility for sub-groups to segregate between patches and for transition between different patterns, even in absence of active agonistic behaviour. The model can be viewed as a network of feedbacks that is independent of the signals or cues involved in mixed groups interactions. Its predictions are therefore applicable to a wide spectrum of situations including social insects (inter castes interaction) and provides insights on possible mechanisms that can be at work.

Aggregation and animal grouping, defined as a higher temporal and spatial density of individuals in some part of space as compared to the surrounding area^1^ represents a prime candidate to understand the role of individual behavioural rules and of the environment constraints in the emergence of collective patterns and social organization.

Many species from bacteria to higher vertebrates form groups more or less stable in time and space in response to environmental heterogeneities and to attraction between individuals^2^. These mutual interactions are mediated by information transfer between group members (signals or cues provided by other individuals) and are at the origin of amplification processes (positive feedbacks) via a variety of mechanisms of visual, mechanical or chemical nature^3^.

Typically, aggregation takes place in patchy environments^2^ where animals tend to gather on resources (e.g. food sources or shelters during resting period^4^). Ordinarily, the process is very fast and the time spent by the individuals outside the patches may be considered as negligible as far as the dynamics is concerned^5^. When many patches are present an uneven distribution of the population among patches (hereafter defined as aggregation) may be the result of inter patches heterogeneities but also, in the case of a set of identical patches, of social interaction between individuals.

So far collective processes have been considered mostly at the species level, where individuals are considered as being similar. In fact the interactions between individuals and the subsequent responses to environmental heterogeneities may vary according to sex, age, size, physiology, personality or strain^6^,^7^,^8^,^9^,^10^,^11^,^12^,^13^,^14^. Furthermore, one also has to account for the possibility that heterospecific interactions^15^,^16^ may be present, the latter being in a sense the extreme case of interindividual differences. In fact mixed-species associations are frequently observed in mammals^17^,^18^, birds^19^,^20^, fish^21^ and arthropods^22^,^23^ but the literature mainly addresses their functionality (e.g., their role against predation)^2^,^17^,^24^ and only few studies are focusing on the mechanisms at their origin^5^.

Many experimental studies and field observations have shown a diversity of heterospecific patterns^25^,^26^,^27^ involving different interaction networks between individuals and between individuals and their environment. In addition to those at work in monospecific aggregation new effects come into play among which segregation, whereby the different populations select different patches, plays a prominent role. Mixed aggregates may then be the result of similar responses to spatial heterogeneities of the different species being tolerant to each other or, in an homogeneous environment, of attraction between species.

Classically, segregation may result from different environmental preferences or from agonistic interaction between species. Several studies showed however that environmental constraints and, in particular, the carrying capacity of patches can play a role in the resulting segregation patterns^5^. In addition of the possibility of being agonistic, neutral or attractive, interactions could also be asymmetrical. For example, studies of chemical communications show that different species can share components (kairormone) to attract each other but that, in some cases, only one species attracts the other and not vice-versa^23^,^28^. Actually, many factors may be at the origin of the patterns reported in these studies but no systematic link between individual-level diversity and the principal characteristics of collective behaviour has been established.

The main objective of the present paper is to show how in an environment composed of identical patches, the diversity of aggregation-segregation patterns is depending on the proportion of different species, the environmental constraints and the heterospecific interactions without the need to change (modulate) the behavioural algorithm of individuals. We build a generic model that integrates the general rules of interattraction between conspecific and heterospecific interactions (in absence of agonistic behaviour) on the one side and the environmental characteristics (sub-groups composition and carrying capacity of the patches) on the other^6^. We show how transitions between different patterns occur in an environment composed of identical patches whose selection cannot result from any heterospecific preferences. We derive a set of coupled differential equations for the model variables and construct the bifurcation diagram of the full set of steady-state solutions. The study is complemented by a stochastic description using Monte Carlo simulations incorporating the effect of fluctuations. We argue that the variety of the behaviours predicted according to three main parameters (sub-group composition, carrying capacity and level of heterospecific interaction) is sufficient to explain in terms of mechanisms the different patterns observed in heterospecific situations. These results can be applied to cases where different strains of the same species interact, or to social insects where different castes are present^4^,^6^,^13^.

## Results

### Model

We start by building a dynamical model of aggregation for two sub-groups of total numbers of individuals *N*_*X*_ and *N*_*Y*_ having to choose between *m* identical patches *i* (*i* = 1, …*m*). Let Ξ_*ji*_ and *Υ*_*ji*_ be the rates at which an individual from respectively subgroups *X* and *Y* leaves the patch *j* for the patch *i*. Experiment shows^29^,^30^ that these rates decrease with the number of individuals already present in the patch in a nonlinear fashion. The particular forms chosen here are:


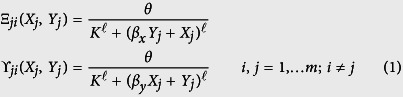


where *K* and 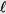 are parameters related to how individuals respond to each other and *β*_*x*_, *β*_*y*_ are interattraction parameters between the two different sub-groups. In the sequel, and by analogy to their shape we will refer to the functions defined by eq. (1) as Hill functions. Finally, *θ* is the maximal speed at which individuals are joining and leaving the patches. In reality, the actual speed at which individuals join a patch *i* is modulated by its carrying capacity and its state of occupation. We account for this effect through the factor


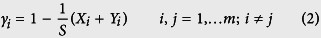


where *S* is the carrying capacity of each patch, being understood that *X*_*i*_ + *Y*_*i*_ ≤ *S*. Actually, the model implicitly imposes that the total population is smaller than or equal to the sum of the carrying capacities of the patches (e.g, total population ≤ *mS*).

The total fluxes of *X* and *Y* individuals between *j* and *i* are thus *γ*_*i*_Ξ_*ji*_*X*_*j*_ and *γ*_*i*_*Υ*_*ji*_*Y*_*j*_ respectively. We may now write the full set of equations for the evolution in time of the number of *X* and *Y* individuals on each patch for the general case of two subgroups *X* and *Y* and for *m* patches as


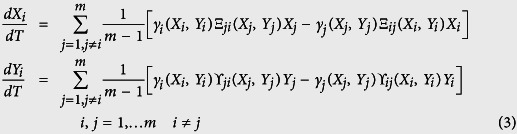


In the sequel, we will choose 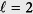 as the Hill exponent parameter, *N*_*x*_ = *N*_*y*_ = *N* (i.e. subgroup populations of equal size) and *m* = 2 (i.e. two patches). After nondimensionalization, eq. (3) reduce to


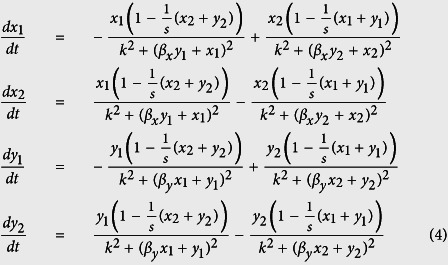


where *x*_*i*_ = *X*_*i*_/*N*, *y*_*i*_ = *Y*_*i*_/*N*, *s* = *S*/*N*, *k* = *K*/*N* and *t* = *θT*/*N*^2^. We notice that *dx*_1_/*dt* + *dx*_2_/*dt* = 0 and *dy*_1_/*dt* + *dy*_2_/*dt* = 0 reflecting that throughout the process the numbers of individuals are conserved (*x*_1_ + *x*_2_ = 1, *y*_1_ + *y*_2_ = 1). Notice that when 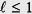, although the rates still decrease with the populations already on the patches, the only solution is the homogeneous one, *x*_*i*_ = *y*_*i*_ = 0.5 (*i* = 1, 2), where the two populations are well-mixed among the two patches (see Supplemental Material A).

**Figure 1:**
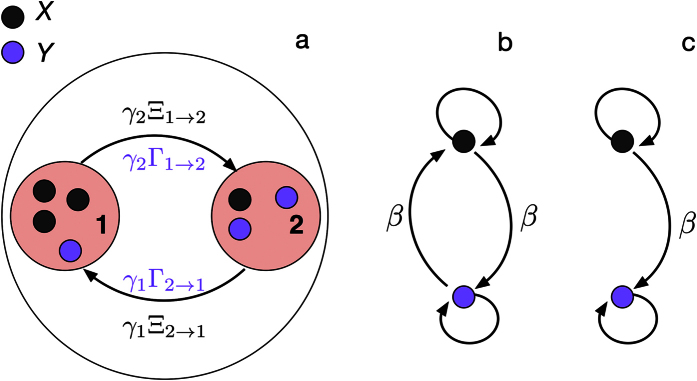
Experimental setup for the study of aggregation/segregation dynamics in an environment containing two equal patches and its relationship with the model defined by eq. (4) (**a**). Positive feedback networks of conspecific and heterospecific interactions : symmetrical (**b**) and asymmetrical (**c**) case.

Figure 1a shows a typical experimental setup corresponding to our theoretical formulation : two patches of a certain carrying capacity are in an arena where individuals of two different sub-groups are evolving. They aggregate under these patches and have a probability to join or to leave each of them. We discard the time spent outside the patches and measure the number of individuals of the two groups on each patch. Figure 1b,c represent in terms of a network the positive feedbacks for the two cases considered in this paper : the case *β*_*x*_ = *β*_*y*_ = *β* (Fig. 1b) and the case *β*_*y*_ = 0, *β*_*x*_ = *β* (Fig. 1c). The negative feedbacks are related to crowding effects and are considered to be identical.

### Symmetrical case: *β*_*x*_ = *β*_*y*_ = *β*

We first consider the simplest scenario where the interattraction parameter *β* acts in the same way for each of the two sub-groups of individuals (i.e. individuals of *x* type are attracted in the same way to *y* individuals as *y* individuals to the *x* ones).

In this case the model defined by eq. (4) possesses four types of solutions of different nature (see Supplemental Material B):

- A homogeneous solution (referred to in the sequel as dispersion) defined by all four variables being equal
- Four semi-homogeneous solutions defined by *x*_1_ = *y*_1_ (referred to in the sequel as aggregation) and by *x*_1_ = *y*_2_ (referred to in the sequel as segregation)
- Four inhomogeneous solutions defined by *x*_1_ ≠ *x*_2_ ≠ *y*_1_ ≠ *y*_2_.

**Figure 2:**
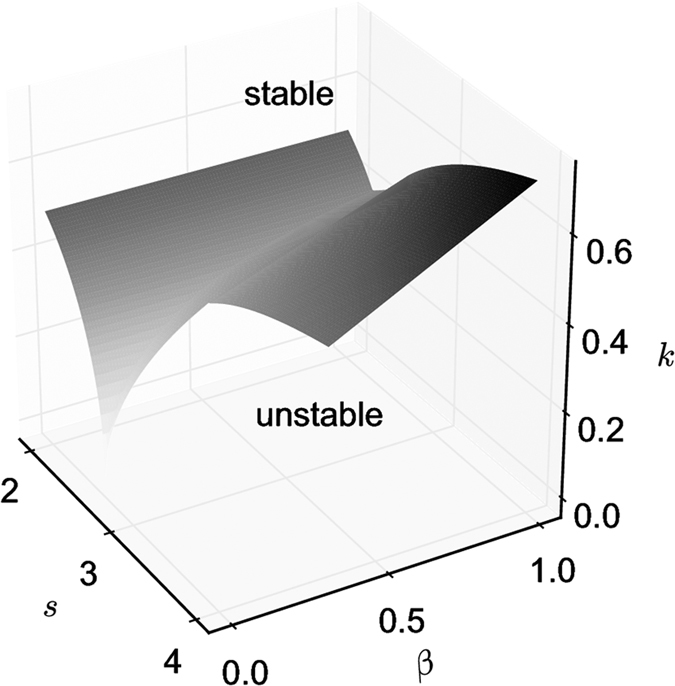
Stability of the dispersion state of model (4) in the case of the symmetrical case *β*_*x*_ = *β*_*y*_ = *β* as a function of the model parameters.

**Figure 3:**
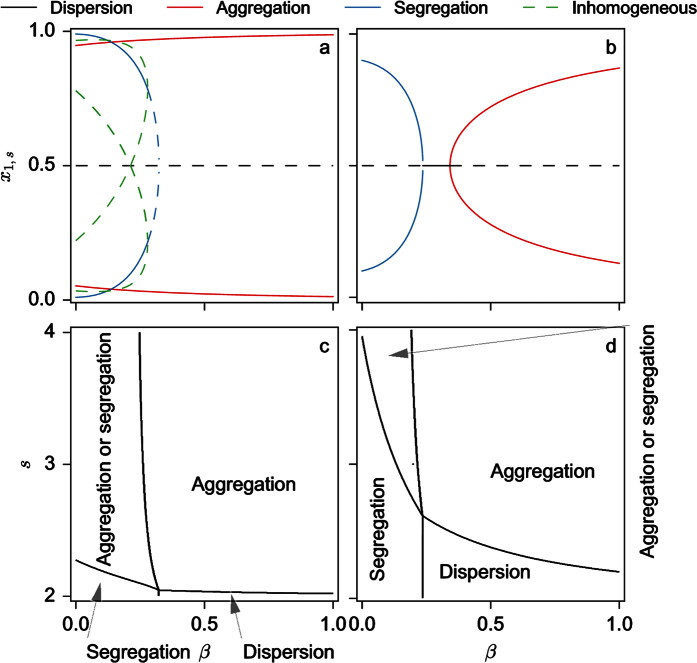
Bifurcation diagrams of the steady states of *x*_1,*s*_ of model (4) as a function of parameter *β* for *s* = 2.5, *k* = 0.1 (**a**) and *k* = 0.3 (**b**). State diagram of the type of existing solutions as a function of *β* and *s* for *k* = 0.1 (**c**) and 0.3 (**d**).

The stability of the nine solutions can also be computed (see Supplemental Material B). Figure 2 shows the region of the full (3-dimensional) parameter space where the homogeneous solution corresponding to the dispersion of the individuals in the two patches is stable or unstable. As seen, the stability of the dispersion state prevails for low values of *s* or high values of *k* and is weakly dependent of *β*. For the other solutions, two examples of bifurcation diagrams for fixed values of *s* and *k* (*s* = 2.5 and *k* = 0.1 and 0.3 respectively) and *β* varying are shown in Fig. 3a,b. As seen, for increasing values of *β*, the system switches from a coexistence between states of aggregation and segregation to a state of aggregation (*k* = 0.1 – Fig. 3a) or from a segregation situation to a dispersion and to an aggregation state (*k* = 0.3 – Fig. 3b).

Figure 3c,d show a more thorough exploration of the parameters in the case of two different values of *k* (*k* = 0.1 and 0.3). For *k* = 0.1 (Fig. 3c) we notice that the segregative state only exists on its own for very small values of *s* and relatively small values of *β* (as long as 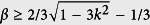; – see Supplemental Material B). Beyond a critical value of *s* and depending on the value of *β*, the system settles either in a state of coexistence between the segregative and aggregative or in a purely aggregative one. The state of dispersion also exists for for a very narrow range of parameter values. The situation qualitatively changes if *k* is taken to be larger (*k* = 0.3, Fig. 3d) : the range of parameters where only segregation occurs and those where only dispersion occurs is larger than the range corresponding to smaller values of *k*.

**Figure 4:**
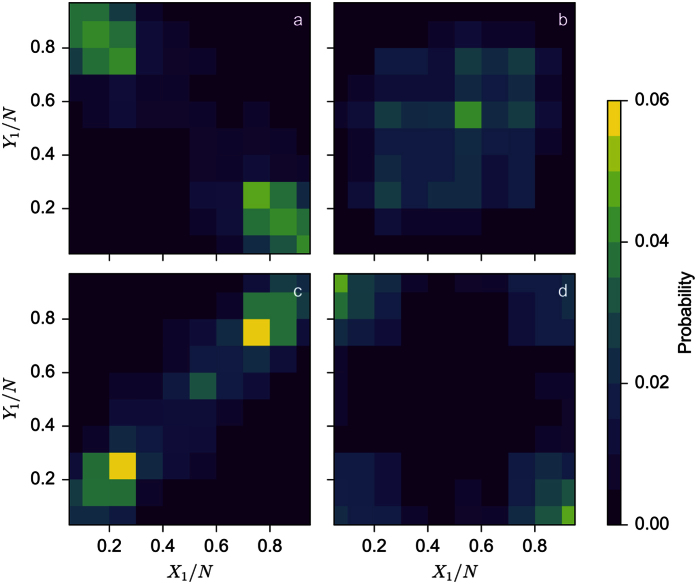
Probability distribution of *X*_1_/*N* and *Y*_1_/*N* as obtained from 10,000 realizations of Monte Carlo simulations for *S* = 2.5*N*, *β* = 0.08 (**a**), *β* = 0.3 (**b**) and *β* = 0.8 (**c**) and for *S* = 6*N* and *β* = 0.08 (**d**). Number of simulation steps is 1000 and other parameters are *N* = 24 and *K* = 0.3*N*.

In order to take into account the noise inherent to the phenomenon we next carry out Monte Carlo simulations. Each process is taken to be as a stochastic process with a certain probability to gain or to lose one unit. We will thus need to adapt model (4) to deal with numbers of units instead of fractions (with *X*_1_ + *X*_2_ = *N*_*x*_ and *Y*_1_ + *Y*_2_ = *N*_*y*_, taking first *N*_*x*_ = *N*_*y*_ = *N*) and to this end we introduce new scaled parameters *S* = *sN*, *K* = *kN* and *T* = *N*^2^*t*. Figure 4a–d display the probability distributions of the fractions *X*_1_/*N* and *Y*_1_/*N* for *N* = 24 and *K* = 0.3*N* and four different parameter values of *S* and *β*. The time of each Monte Carlo simulation is fixed to 1000 steps and the number of realisations is 10,000. In Fig. 4a (*S* = 2.5*N*, *β* = 0.08), we see that we have two peaks centered on (0, 1) and (1, 0) corresponding to the segregation situation. When *S* = 2.5*N* and *β* = 0.3 (Fig. 4b) the probability distribution is now centred on (0.5, 0.5) reflecting the stability of the dispersion state. The situation changes radically in Fig. 4c (*S* = 2.5*N*, *β* = 0.8) where the probability distribution has now two peaks centred on (0, 0) and (1, 1) corresponding to individuals of both sub-groups aggregating together in patch 1 or 2. Finally, Fig. 4d (*S* = 6*N*, *β* = 0.08) shows the probability distribution in the case where individuals either aggregate or segregate.Note that the variability of these distributions is not negligible and that states that are not predicted by the mean field formulation are visited. However, the results of the mean-field approximation and the averages over many realizations of the Monte Carlo simulation are in total agreement.

The results presented so far deal with the case *N*_*x*_ = *N*_*y*_. We now consider distribution of individuals when *N*_*x*_ ≠ *N*_*y*_. Indeed, a non trivial case is provided by a situation where a patch is not capable on its own to host all individuals of both sub-groups. In order to explore this case we ran simulations where we varied the number of individuals in the two sub-groups, while the carrying capacity *S* was the same. We used two different indexes to measure the degree upon which individuals are segregating or aggregating. The first is the Duncan index defined as^31^


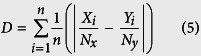


which takes the values of 0 if all individuals are aggregated or dispersed and 1 if all individuals are segregated. The second, complementary measure we take is the entropy defined as^32^


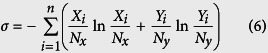


which now takes the value of 0 if individuals are segregated or aggregated and 2 ln 2 if the system is well-mixed. Note that many other indexes of segregation or dissimilarity exist in the literature (see e.g. ref. 33).

**Figure 5:**
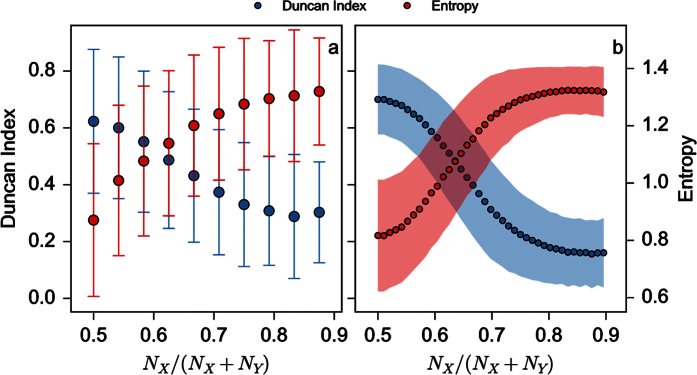
Mean and standard deviation of the Duncan index and of the entropy (eqs (5) and (6) respectively) as a function of the proportion *N*_*X*_/(*N*_*X*_ + *N*_*Y*_) for *N*_*X*_ + *N*_*Y*_ = 24 and *S* = 13 (**a**) and *N*_*X*_ + *N*_*Y*_ = 96 and *S* = 49 (**b**). Other parameter values are *β* = 0.02, *k* = 1/3(*N*_*X*_ + *N*_*Y*_)/2. Number of steps per simulations is 1000 and number of realizations is 10,000.

Figure 5a,b shows the evolution of the Duncan index and the entropy as a function of the proportion of individuals of *X* type. We start with the same number of *X* and *Y* individuals (12, or 48 corresponding to *N*_*X*_/(*N*_*X*_ + *N*_*Y*_) = 0.5) and then remove and add individual of *Y* and *X* type respectively while keeping the total population *N*_*X*_ + *N*_*Y*_ constant (here 24 (Fig. 5a) and 96 (Fig. 5b)). As seen in the figures, both indexes display an inflexion point, which is more marked for high values of *N*_*X*_ + *N*_*Y*_, separating the segregative regime from the dispersion or partly homogeneous one, showing how an inhomogeneity in the number of individuals in the two sub-groups favors aggregation or homogeneity. Notice that when the individuals are simply distributed randomly in the two patches over 10,000 realizations, the mean and standard deviation of the Duncan index and the entropy are nearly constant as a function of *N*_*X*_/(*N*_*X*_ + *N*_*Y*_) (~0.34 ± 0.24 and ~1.02 ± 0.25 respectively – not shown).

### Asymmetrical case: *β*_*x*_ = *β*, *β*_*y*_ = 0

We next explore the situation where one sub-group is attracted to the other *β*_*x*_ = *β* while the other is not *β*_*y*_ = 0. This situation is not fully accessible analytically, except for the homogeneous solution. Still, by combining the first and third equations of the model (4) we were nevertheless able to cast the problem to a ninth degree algebraic equation (see Supplemental Material C).

**Figure 6:**
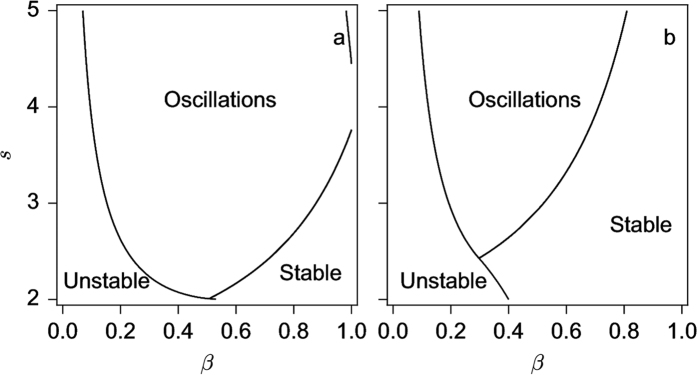
Stability of the homogeneous state of model (4) in the case of asymmetrical case *β*_*x*_ = *β* and *β*_*y*_ = 0 as a function of *β* and *s* for *k* = 0.1 (**a**) and *k* = 0.3 (**b**).

The nature of the solutions remains basically the same as in the previous case. For some range of parameter values one also has the possibility of oscillatory behaviour around the homogeneous state. Figure 6a,b depict the stability around the homogeneous state for two values of parameter *k* (*k* = 0.1 and 0.3). As seen the range of parameters where the homogeneous state displays oscillations decreases as *k* increases. On inspecting the bifurcation diagrams for these *k* values and fixing *s* = 2.8, we see

**Figure 7:**
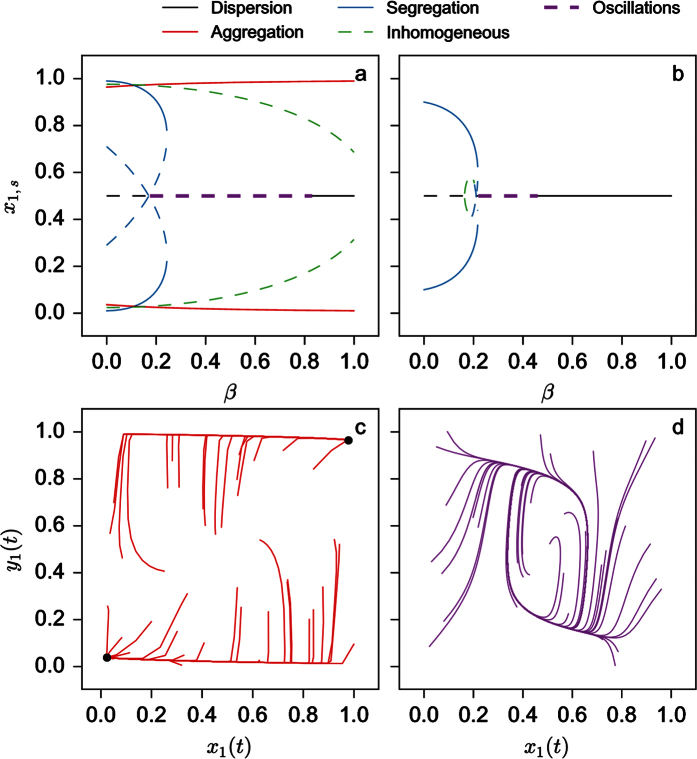
Bifurcation diagrams of the steady states of *x*_1,*s*_ of the model (4) as a function of parameter *β* for *s* = 2.8, *k* = 0.1 (**a**) and *k* = 0.3 (**b**); Numerical integration of the equations of the model (4) for *s* = 2.8, *β* = 0.35 and *k* = 0.1 (**c**) and *k* = 0.3 (**d**).

- For Fig. 7a: we start for small *β* by having a coexistence between segregative and aggregative state. When the state corresponding to segregation loses its stability, the aggregative state coexists with the homogeneous state that displays oscillations. In practice, we never observe these oscillations (see numerical integrations of the model for 40 random initial conditions in Fig. 7c – *β* = 0.35), meaning that the attraction basin is very small. Finally, for large *β* only aggregation is possible (*k* = 0.1, Fig. 7a).
- For Fig. 7b: for small *β*, only segregation occurs until the corresponding solution becomes unstable. Then we observe only oscillations (Fig. 7d – *β* = 0.35) around the homogeneous state until a second critical parameter value where the homogeneous state is again stable and is the only one to exist.

**Figure 8:**
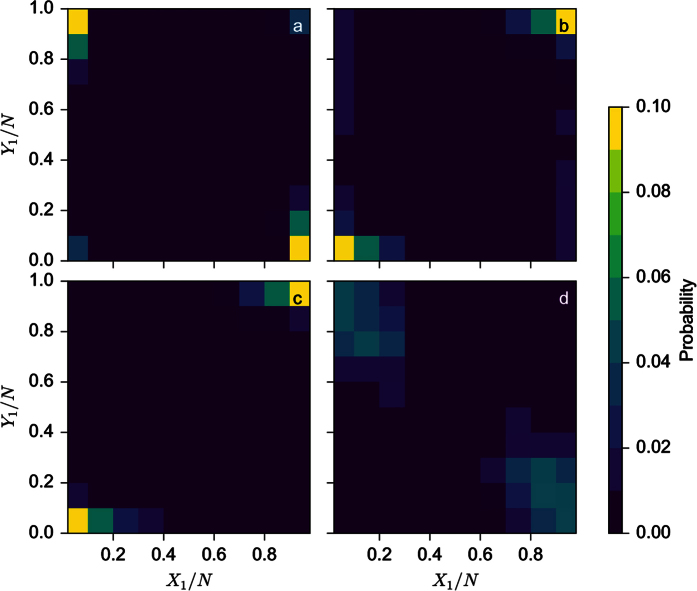
Probability distribution of *X*_1_/*N* and *Y*_1_/*N* as obtained from 10,000 realizations of Monte Carlo simulations for *K* = 0.1*N*, *β* = 0.1 (**a**), *β* = 0.3 (**b**) and *β* = 0.8 (**c**) and for *K* = 0.3*N* and *β* = 0.08 (**d**). Number of simulation steps is 1000 and other parameters are *N* = 24 and *S* = 0.3*N*.

As in the previous subsection, we now perform Monte Carlo simulations. Four examples of probability distribution are shown in Fig. 8a–d. We fix *N* = 24 and *S* = 3*N* and vary *K* and *β*. For *K* = 0.1*N* and *β* = 0.1 (Fig. 8a), we see four peaks in the probability distribution corresponding to the coexistence between aggregative and segregative state. As *β* increases (*β* = 0.3, 0.8, *k* = 0.1*N* - Fig. 8b,c, respectively), we see that aggregation occurs more and more often. Another example is provided in Fig. 8d, where for a larger value of *K* and a small value of *β* (*K* = 0.3*N*, *β* = 0.1) the segregative state is more probable.

## Discussion

We developed in this paper a mathematical model for aggregation and segregation of two sub-groups having to choose among two patches accounting for conspecific and heterospecific interactions and, more generally, for interactions between different sub-groups (species, strains, castes in social insects, *etc.*). We have identified a wealth of non-trivial collective behaviours, without any change of individuals’ algorithm. More specifically, by taking into account the composition of each sub-group (*N*_*X*_ and *N*_*Y*_), the environmental parameter corresponding to the carrying capacity (*S*) and the strength and the reciprocity of the inter-attraction between individuals from different sub-groups (*β*), we showed the conditions under which collective behaviours of different nature could be observed. The analysis of the model was limited to two sub-groups confronted to an environment composed by two patches of given carrying capacity. For an equal number of individuals in each sub-group and a reciprocal inter-attractivity (*X* is attracted to *Y* with the same strength than *Y* is attracted to *X* – *β*_*X*_ = *β*_*Y*_ = *β*), we were able to compute all the existing solutions at the steady-state and to identify critical parameter values beyond which a particular behaviour could be observed. Aggregation (*X*_*i*_ = *Y*_*i*_, *i* = 1 or 2) in one patch could for example be observed if *S* and *β* are large enough. On the contrary, for small values of these parameters (below critical values) aggregation disappears and segregation between the two subgroups (*X*_*i*_ = *Y*_*j*_, *i*, *j* = 1, 2 and *j* ≠ *i*) takes place. For intermediate values of these parameters, either a dispersion occurs (*X*_1_ = *X*_2_ = *Y*_1_ = *Y*_2_, *S* < *S*_*c*_ and *β* > *β*_*c*_) or a multi stable regime where aggregation or segregation are observed with a certain probability (*S* > *S*_*c*_ and *β* < *β*_*c*_). Stochastic (Monte Carlo) simulations confirms the behavioural patterns obtained by model (4). Next, we analysed the situation where *N*_*X*_ > *N*_*Y*_ while *N*_*X*_ + *N*_*Y*_ and *S* were kept constant. A surprising result was that introducing an heterogeneity in the population sub-groups favors an homogeneous (dispersion) distribution of the different individuals. A last case study concerned asymmetric inter-attractions (*β*_*X*_ = *β*, *β*_*Y*_ = 0) for equal population sizes : we showed that while still having qualitatively behaviours of the same nature there exists, for a certain range of parameters, a situation where the system is not able to stabilise and performs oscillations around the homogeneous state (Fig. 7d).

Many studies devoted to heterospecific interactions have been reported, from unicellular and social amoeba^34^ to woodlices^35^, ants^36^,^37^ and mammals^2^,^20^,^38^. For example, some species of Triatominae are attracted to the chemical signals left from individuals of the same and of different species^39^. At the same time, in a binary choice experiment involving one or two species, individuals are not able to aggregate (or segregate) in a particular shelter and display therefore a spatial dispersion (equal distribution of individuals between the two shelters)^40^. Our model accounts for these situations even though the feedbacks retained relate to conspecific and heterospecific attractions. In fact, the model predicts a range of parameters where one can observe this dispersion either by setting the Hill parameter 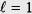 or by having a low response threshold *K*. Furthermore, despite the fact that no agonistic mechanism is incorporated in the model, we predict that in some range of parameters segregation is happening as a consequence of the limited size of the carrying capacity. It is worth noting that the model analysed and the results obtained are largely independent of the particular types of signals or cues used by different species. Indeed, the model can be viewed as a network of feedbacks between individuals of the same sub-group and of different sub-groups whose presence or absence and strength determines the behaviours observed. Without denying the importance of the specificities of each of the cases encountered in nature, the model is therefore generic and applicable to a whole spectrum of situations involving different feedbacks. In fact, one can predict the global patterns generated by all possible feedback networks involving two sub-groups, for given negative feedbacks (here related to the carrying capacities). Table 1 describes all possible outcomes in the cases where positive feedbacks between conspecifics and heterospecifics are present or absent (column 1) for a non or weak (column 2) and for a strong (column 3) inter-attraction. In this respect it is interesting to note that within our framework the condition to observe segregative behaviour is to have also aggregation within conspecifics the other way being to have agonistic mechanisms, which are absent in our model. All in all, the merit of such a model is to provide a unifying framework from which very diverse situations can be addressed and to give hints on the mechanisms at the basis of a phenomenon observed during an experiment involving two species or strains.

An important parameter in our model is the inter-attraction parameter *β*. Inter-attraction between species^28^,^41^ or strains^6^ has been highlighted in many taxa. For example, it has been shown that two species of cockroaches (*P. americana* and *P. fuliginosa*) may either segregate or aggregate depending on the number of individuals on each sub-groups when given the choice between two shelters^5^. Our model (eq. (4)) is in full agreement with this experiment. Other examples are known in the context of functional duality of aggregation substances in cockroaches^42^ and bark beetles^43^. As far as chemical communications between species are concerned, the strength of our inter-attraction parameter may be related to the percentage of molecules shared by the two species. Actually, a natural hypothesis is that individuals of the different species may cooperate if they share a number of common “linguistic” components (0 < *β* < 1) or if they “speak the same language” (*β* = 1). Different chemical analyses describe such common components (e.g. for stink bugs^44^ or for locusts^45^) or show the ability to respond to the chemical compounds of another species. There is also evidence that in ant colonies, although individuals from different castes (e.g. foragers and domestics) share the same type of chemicals, different chemical signatures exist betwen them^46^. Subtle differences may therefore be present at the level of cross-attraction favoring a particular spatial organization between these castes. *A contrario* it has been shown that hydrocarbon profiles of two species of cockroaches are superimposed when put in heterospecific conditions^47^. This suggests that the inter-attraction parameter could then increase and facilitate aggregation until no heterospecific differences exist between individuals (e.g., *β* = 1).

We also addressed results in the case of asymmetrical inter-attraction between sub-groups. Although more rarely reported, its presence has been suggested in different species belonging to the genus *Forficula* of the Order Dermaptera^23^. It would be interesting to perform additional experiments on these species in the setup similar to that of Fig. 1a in order to confront our predictions and, in particular, to observe an oscillatory regime (Fig. 7b,d).

By its genericity, the model developed in this paper can be extended in many ways to explore a variety of cases that can be directly tested in laboratory. A first question is whether the solutions and the behaviours predicted are of the same nature in the presence of asymmetrical set-up (e.g., patches of different size), or of a totally homogeneous environment (an arena without patches). Even in the case where one ends up with one aggregate, one could wonder whether this aggregate is well mixed or if there is some level of segregation between individuals of different sub-groups. In this context, different theoretical studies predict the emergence of segregation within a single aggregate^48^,^49^. A different extension would be to account for the fact that aggregation phase is happening during the day (e.g, in cockroaches). At night, individuals become more active and forage. They are moving then from patch to patch and the spatial distribution – be it aggregation, segregation or dispersion – gained during the day is disturbed. The question is then whether in an heterospecific experiment individuals are going back to their original distribution or if a small change in the individuals’ number on each patch is sufficient to lead to a completely different distribution the next day^50^. It would also be worth to revisit our results and predictions in the light of symbiotic relationships between species and their functionality^51^, be they mutualism – reflected in our model by *β*_*X*_ = *β*_*Y*_ = *β* or commensalism – reflected by *β*_*X*_ = *β* and *β*_*Y*_ = 0. Indeed a classical idea is that aggregation within a species may result in empty patches leaving space for other species to settle, and therefore to segregation^52^. A question would therefore be to what extent inter-attraction between individuals of different sub-groups affects the spatial organization and whether the subsequent Allee effects ought to be revisited in the context of heterospecificity.

Finally, increasing the number of patches would undoubtedly bring the model closer to real-world situations. Using this number as a bifurcation parameter, more and more solutions of new types will be generated corresponding to inhomogeneous stable states of aggregation/segregation. Preliminary results suggest that these patterns are associated to situations where only a subset of patches is clearly occupied, the remaining ones being almost empty. Depending on the sizes of the populations and the strength of the interattraction, these occupied patches correspond to states of dispersion or segregation.

## Additional Information

**How to cite this article**: Nicolis, S. C. *et al*. Transition between segregation and aggregation: the role of environmental constraints. *Sci. Rep.* **6**, 32703; doi: 10.1038/srep32703 (2016).

## Supplementary Material

Supplementary Information

## Figures and Tables

**Table 1:**
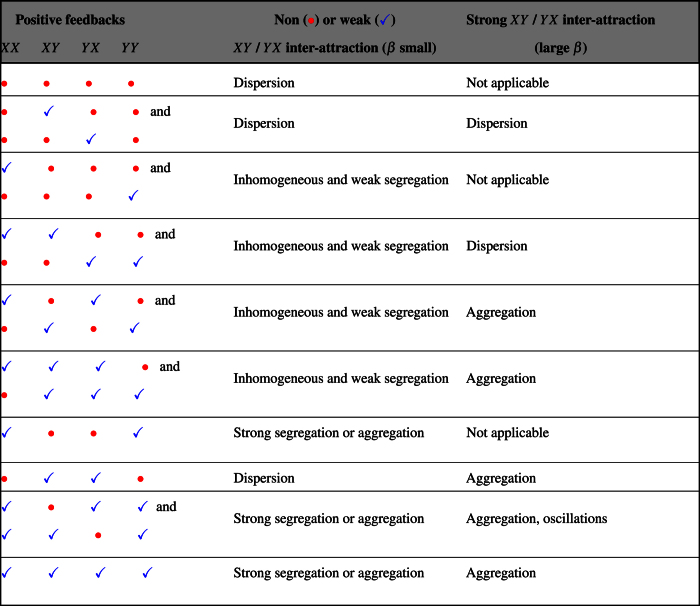
Types of feedbacks that may be present (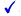) or absent (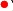) (column 1) and the corresponding predicted behaviour of our model in the cases of non or weak (column 2) and of strong (column 3) inter-attraction.

"Inhomogeneous" defines a case where the total population is unevenly distributed among the two patches.
