# Supplementary material for: Transition between segregation and aggregation: the role of environmental constraints
Source: Sci Rep. 2016 Sep 7;6:32703. doi: 10.1038/srep32703 (PMC5013323; doi:10.1038/srep32703)
Supplement: Supplementary Information [file srep32703-s1.pdf]

Transition between segregation and aggregation : the role of environmental constraints
Supplemantary Material : Resolution of the system of equations (4)

S. C. Nicolis, J. Halloy and J-L Deneubourg

A Symmetrical case : $\beta_x = \beta_y = \beta, \ell = 1$

After some straightforward manipulations, the first and third equations of model (4) at the steady state lead to

$$\begin{aligned} -x_1\left(k+x_2+\beta y_2\right)\left(s-x_2-y_2\right)+x_2\left(k+x_1+\beta y_1\right)\left(s-x_1-y_1\right) &= 0 \\ -y_1\left(k+y_2+\beta x_2\right)\left(s-x_2-y_2\right)+y_2\left(k+y_1+\beta x_1\right)\left(s-x_1-y_1\right) &= 0 \end{aligned} \tag{A.1}$$

Keeping in mind that $x_2y_2 - x_1y_2 = y_1 - x_1$ because of conservation of $x_1 + x_2$ and $y_1 + y_2$ we have

$$\begin{aligned} &ks\left(x_2-x_1\right)+x_1x_2\left(y_2+x_2-x_1-y_1\right)-\left(\beta s-k\right)\left(x_1-y_1\right)+ \\ &\left(x_1x_2y_2+x_1y_2^2-x_2x_1y_1-x_2y_1^2\right)\beta=0 \end{aligned} \tag{A.2}$$

$$\begin{aligned} &ks\left(y_2-y_1\right)+y_1y_2\left(y_2+x_2-x_1-y_1\right)+\left(\beta s-k\right)\left(x_1-y_1\right)+ \\ &\left(y_1y_2x_2+y_1x_2^2-y_2y_1x_1-y_2x_1^2\right)\beta=0 \end{aligned} \tag{A.3}$$

Adding together the two equations (A.2) and (A.3) yields after some rearrangements,

$$2\underbrace{\left(1-x_1-y_1\right)}_{T_1}\underbrace{\left(\beta x_1y_2+x_1x_2+\beta x_2y_1+ks+y_1y_2\right)}_{T_2}=0 \tag{A.4}$$

Clearly, the second factor T_2 of equation (A.4) cannot lead to a solution, as all terms are positive. We are therefore left with $T_1 = 0$, i.e.

$$y_1 = 1 - x_1 \qquad \text{and similarly} \qquad y_2 = 1 - x_2 \tag{A.5}$$

Substituting equation (A.5) into equation (A.2), we obtain

$$\left(x_1-x_2\right)\left(s-1\right)\left(k+b\right)=0 \tag{A.6}$$

and thus

$$x_1 = x_2 = y_1 = y_2 = \frac{1}{2} \tag{A.7}$$

B Symmetrical case : $\beta_x = \beta_y = \beta, \ell = 2$

B.1 Steady states and stability

B.1.1 Homogeneous solution ($x_1 = x_2 = y_1 = y_2$)

Equating all variables of eqs. (4) gives straightforwardly :

$$x_{1,s} = x_{2,s} = y_{1,s} = y_{2,s} = 0.5 \tag{B.1}$$

corresponding to the case where the individuals of each subgroup equally select both patches, signaling a situation of dispersion

B.1.2 Aggregation ($x_1 = y_1$)

Setting $x_1 = y_1 = x$ and $x_2 = 1 - x$, $y_2 = 1 - y_1$ and eliminating the homogeneous solution $x = 1/2$ already found we are left with

$$x^2 - x + \delta_1 = 0 \quad \text{where} \quad \delta_1 = \frac{k^2 s}{(\beta+1)^2(s-2)}$$

This equation has two solutions :

$$x_{1,s} = y_{1,s} = \frac{1}{2}\left(1 \pm \sqrt{1 - 4\delta_1}\right) \tag{B.2}$$

which exist as long as $\delta_1 \leq 1/4$, or

$$\beta \geq \frac{1}{s-2}\left(-s \pm 2\sqrt{k^2 s\,(s-2)} + 2\right) \tag{B.3}$$

B.1.3 Segregation ($x_1 = y_2$).

Setting this time $x_1 = y_2 = x$ and $x_2 = 1 - x$, $y_1 = 1 - x$ we have

$$x^2 - x + \delta_2 = 0 \quad \text{where} \quad \delta_2 = \frac{k^2 + \beta^2}{(\beta-1)^2}$$

This equation has two solutions :

$$x_{1,s} = y_{2,s} = \frac{1}{2}\left(1 \pm \sqrt{1 - 4\delta_2}\right) \tag{B.4}$$

which exist as long as $\delta_2 \leq 1/4$, or

$$\beta \geq \frac{2}{3}\sqrt{1 - 3k^2} - \frac{1}{3} \tag{B.5}$$

B.1.4 Inhomogeneous solutions($x_1 \neq x_2 \neq y_1 \neq y_2$).

Adding and subtracting the first and third equations of eqs. (4) at the steady state we have

$$(-x_1 - y_1 + 1)\Big(\beta^2 s\,(2x_1y_1 - x_1 - y_1) + \beta^2\left(x_1^2 - 6x_1y_1 + 2x_1 + y_1^2 + 2y_1\right) + \beta s\,(4x_1y_1 - 2x_1 - 2y_1) + \beta\left(-2x_1^2 - 4x_1y_1 + 4x_1 - 2y_1^2 + 4y_1\right) + s\left(2k^2 + 2x_1^2 - 2x_1y_1 - x_1 + 2y_1^2 - y_1\right) - 3x_1^2 + 2x_1y_1 + 2x_1 - 3y_1^2 + 2y_1\Big) = 0$$

$$(x_1 - y_1)\Big(\beta^2 s\,(2x_1y_1 - x_1 - y_1 - 1) + \beta^2\left(x_1^2 - x_1 + y_1^2 - y_1 + 2\right) + \beta s\,(-4x_1y_1 + 2x_1 + 2y_1 - 2) + \beta\left(2x_1^2 + 8x_1y_1 - 6x_1 + 2y_1^2 - 6y_1 + 4\right) + s\left(-2k^2 - 2x_1^2 - 2x_1y_1 + 3x_1 - 2y_1^2 + 3y_1 - 1\right) + 3x_1^2 + 4x_1y_1 - 5x_1 + 3y_1^2 - 5y_1 + 2 + 2k^2\Big) = 0 \tag{B.6}$$

We notice that

- We can factor out the solutions $x_1 = 1 - y_1 = y_2$ and $x_1 = y_1$ found earlier.
- Upon the change of variables, $x_1 + y_1 = u$, $x_1y_1 = v$, equation (B.6) reduces to

$$\begin{aligned} v_s &= \frac{1}{2}\frac{\alpha_1 u^2 + \alpha_2 u - 2k^2}{\alpha_3} \\ 0 &= u^2 - 2u + \alpha_4 \quad \rightarrow u_s = 1 \pm \sqrt{1 - \alpha_4} \end{aligned} \tag{B.7}$$

where

$$\alpha_1 = -\beta^2 + 2\beta - 2s + 3, \quad \alpha_2 = (\beta + 1)^2\,(s - 2)\,, \quad \alpha_3 = (\beta - 1)\,(\beta s - 4\beta + 3s - 4)$$
$$\alpha_4 = \frac{(s-2)\left(\beta^2\,(s-4) + \beta\,(4s-8) + 4k^2 s - 4k^2 + 3s - 4\right)}{(\beta - 2s + 3)\,(3\beta - 2s + 3)}$$

Therefore, switching back to the original variables x_1 and y_1 we have

$$\begin{aligned} x_{1,s}y_{1,s} &= \frac{1}{2}\frac{\alpha_1 u^2 + \alpha_2 u - 2k^2}{\alpha_3} \\ x_{1,s} + y_{1,s} &= 1 \pm \sqrt{1 - \alpha_4} \end{aligned} \tag{B.8}$$

which can be straightforwardly solved.

Now that we have analytical expressions of the different types of solutions, we need to evaluate their stability. As stated earlier, the model possesses four variables but because there is conservation, we are left with two equations for e.g. x_1 and y_1. Evaluation of the elements of the Jacobian matrix leads to the characteristic equation :

$$\lambda^2 + \epsilon_1 \lambda + \epsilon_2 = 0 \tag{B.9}$$

where

$$\begin{aligned}
\epsilon_1 = \quad & -\frac{2A_1A_5}{\left(A_5^2+k^2\right)^2}\left(-\frac{A_0}{s}+1\right)+\frac{A_1}{s\left(A_5^2+k^2\right)}-\frac{2A_2A_6}{\left(A_6^2+k^2\right)^2}\left(-\frac{A_0}{s}+1\right)+ \\
& \frac{A_2}{s\left(A_6^2+k^2\right)}-\frac{2A_3x_1}{\left(A_3^2+k^2\right)^2}\left(1-\frac{1}{s}\left(-A_0+2\right)\right)-\frac{2A_4y_1}{\left(A_4^2+k^2\right)^2}\left(1-\frac{1}{s}\left(-A_0+2\right)\right)+ \\
& \frac{1}{A_4^2+k^2}\left(1-\frac{1}{s}\left(-A_0+2\right)\right)+\frac{1}{A_3^2+k^2}\left(1-\frac{1}{s}\left(-A_0+2\right)\right)+ \\
& \frac{-\frac{A_0}{s}+1}{A_6^2+k^2}+\frac{-\frac{A_0}{s}+1}{A_5^2+k^2}+\frac{x_1}{s\left(A_3^2+k^2\right)}+\frac{y_1}{s\left(A_4^2+k^2\right)} \\
\epsilon_2 = \quad & -\left[\frac{2A_1A_5\beta}{\left(A_5^2+k^2\right)^2}\left(-\frac{A_0}{s}+1\right)-\frac{A_1}{s\left(A_5^2+k^2\right)}+\frac{2A_3\beta x_1}{\left(A_3^2+k^2\right)^2}\left(1-\frac{1}{s}\left(-A_0+2\right)\right)-\right. \\
& \left.\frac{x_1}{s\left(A_3^2+k^2\right)}\right]\left[\frac{2A_2A_6\beta}{\left(A_6^2+k^2\right)^2}\left(-\frac{A_0}{s}+1\right)-\frac{A_2}{s\left(A_6^2+k^2\right)}+\right. \\
& \left.\frac{2A_4\beta y_1}{\left(A_4^2+k^2\right)^2}\left(1-\frac{1}{s}\left(-A_0+2\right)\right)-\frac{y_1}{s\left(A_4^2+k^2\right)}\right]+\left[\frac{2A_1A_5}{\left(A_5^2+k^2\right)^2}\left(-\frac{A_0}{s}+1\right)-\right. \\
& \frac{A_1}{s\left(A_5^2+k^2\right)}+\frac{2A_3x_1}{\left(A_3^2+k^2\right)^2}\left(1-\frac{1}{s}\left(-A_0+2\right)\right)+\frac{1}{A_3^2+k^2}\left(-1+\frac{1}{s}\left(-A_0+2\right)\right)- \\
& \left.\frac{-\frac{A_0}{s}+1}{A_5^2+k^2}-\frac{x_1}{s\left(A_3^2+k^2\right)}\right]\left[\frac{2A_2A_6}{\left(A_6^2+k^2\right)^2}\left(-\frac{A_0}{s}+1\right)-\frac{A_2}{s\left(A_6^2+k^2\right)}+\right. \\
& \frac{2A_4y_1}{\left(A_4^2+k^2\right)^2}\left(1-\frac{1}{s}\left(-A_0+2\right)\right)+\frac{1}{A_4^2+k^2}\left(-1+\frac{1}{s}\left(-A_0+2\right)\right)- \\
& \left.\frac{-\frac{A_0}{s}+1}{A_6^2+k^2}-\frac{y_1}{s\left(A_4^2+k^2\right)}\right]
\end{aligned}$$

and

$$\begin{aligned}
& A_0 = x_1 + y_1, \quad A_1 = 1 - x_1, \quad A_2 = 1 - x_2, \\
& A_3 = \beta y_1 + x_1, \quad A_4 = \beta x_1 + y_1, \\
& A_5 = \beta(1-y_1) - x_1 + 1 \quad A_6 = \beta(1-x1) - y_1 + 1
\end{aligned}$$

Replacing then the stationary solutions $x_{i,s}, y_{i,s}$ $(i = 1, 2)$ into equation (B.9), we are able to assess the stability of the solutions of different nature found. In particular, the homogeneous state is analytically accessible because of its explicit expression. In that case, the associated eigenvalues read

$$\begin{aligned} \lambda_1 &= -\frac{8\,(s-1)\left(3\beta^2+2\beta+4k^2-1\right)}{s\left(\beta^2+2\beta+4k^2+1\right)^2} \\ \lambda_2 &= \frac{8\left(\beta^2 s-2\beta^2+2\beta s-4\beta-4k^2 s+s-2\right)}{s\left(\beta^2+2\beta+4k^2+1\right)^2} \end{aligned} \tag{B.10}$$

The condition for the homogeneous state to be stable is that the real parts of the two eigenvalues are negative. We notice that the common denominator is always positive. We then have the following conditions

$$\begin{aligned} k &> \pm\frac{\sqrt{-(\beta+1)(3\beta-1)}}{2} \\ k &> \pm\frac{\sqrt{\frac{(\beta+1)^2(s-2)}{s}}}{2} \end{aligned} \tag{B.11}$$

C Asymmetrical case : $\beta_x = \beta$ and $\beta_y = 0, \ell = 2$

This situation is not fully accessible analytically, but by combining the first and third equations of the model (4) we are nevertheless able to cast the problem to the following ninth degree algebraic equation and the following relation between x_1 and y_1

$$\begin{aligned} &\left(y_1-\frac{1}{2}\right)\left(\chi_8 y_1^8+\chi_7 y_1^7+\chi_6 y_1^6++\chi_5 y_1^5++\chi_4 y_1^4++\chi_3 y_1^3++\chi_2 y_1^2++\chi_1 y_1+\chi_0\right)=0 \\ x_1 &= \frac{\left(-2k^2 s y_1+k^2 s+k^2 y_1-2 s y_1^3+3 s y_1^2-s y_1+3 y_1^3-5 y_1^2+2 y_1\right)}{k^2-y_1^2+y_1} \end{aligned} \tag{C.1}$$

where

$$\begin{aligned}
\chi_8 &= (-s+2)(\beta-2s+3)(\beta+2s-3) \\
\chi_7 &= (4s-8)(\beta-2s+3)(\beta+2s-3) \\
\chi_6 &= \beta^2\left(-3k^2s+3k^2-6s+12\right)+\beta\left(-8k^2s^2+24k^2s-18k^2\right)+ \\
&\quad 12k^2s^3-48k^2s^2+60k^2s-22k^2+25s^3-125s^2+206s-112 \\
\chi_5 &= \beta^2\left(9k^2s-9k^2+4s-8\right)+\beta\left(24k^2s^2-72k^2s+54k^2\right)- \\
&\quad 36k^2s^3+144k^2s^2-180k^2s+66k^2-19s^3+95s^2-156s+84 \\
\chi_4 &= \beta^2\left(k^4s-4k^4-10k^2s+11k^2-s+2\right)+\beta\Bigg(-16k^4s^2+32k^4s-12k^4- \\
&\quad 26k^2s^2+78k^2s-58k^2\Bigg)+12k^4s^3-36k^4s^2+30k^4s-6k^4+39k^2s^3-156k^2s^2+ \\
&\quad 195k^2s-72k^2+7s^3-35s^2+57s-30 \\
\chi_3 &= \beta^2\left(-2k^4s+8k^4+5k^2s-7k^2\right)+\beta\left(32k^4s^2-64k^4s+24k^4+12k^2s^2-36k^2s+26k^2\right)- \\
&\quad 24k^4s^3+72k^4s^2-60k^4s+12k^4-18k^2s^3+72k^2s^2-90k^2s+34k^2-s^3+5s^2-8s+4 \\
\chi_2 &= k^2\Bigg(\beta^2\left(3k^4s-k^4+k^2s-6k^2-s+2\right)+\beta\Bigg(-8k^4s^2+8k^4s-2k^4-20k^2s^2+40k^2s- \\
&\quad 16k^2-2s^2+6s-4\Bigg)+4k^4s^3-8k^4s^2+4k^4s-2k^4+15k^2s^3-45k^2s^2+ \\
&\quad 38k^2s-8k^2+3s^3-12s^2+15s-6\Bigg) \\
\chi_1 &= -k^4\Bigg(\beta^2\left(3k^2s-k^2-2\right)+\beta(-8k^2s^2+8k^2s-2k^2-4s^2+8s-4)+ \\
&\quad 4k^2s^3-8k^2s^2+4k^2s-2k^2+3s^3-9s^2+8s-2\Bigg) \\
\chi_0 &= k^6s\left(\beta^2+\beta(-2s+2)+k^2+s^2-2s+1\right) \qquad \text{(C.2)}
\end{aligned}$$

Apart from the homogeneous solution which is directly accessible, equations (C.1) and (C.2) can only be solved numerically.
